# Abscopal Effect after Radiosurgery for Solitary Brain Metastasis from Non-small Cell Lung Cancer

**DOI:** 10.7759/cureus.3777

**Published:** 2018-12-26

**Authors:** Andrew J Hamilton, Jerome Seid, Kyle Verdecchia, Paul Chuba

**Affiliations:** 1 Family Medicine, Ascension Macomb-Oakland, Madison Heights, USA; 2 Oncology, Ascension Macomb-Oakland, Warren, USA; 3 Radiation Oncology, Ascension Macomb-Oakland, Rochester Hills, USA; 4 Radiation Oncology, Ascension Macomb-Oakland, Warren, USA

**Keywords:** abscopal effect, radiosurgery, radiotherapy, non-small cell lung cancer, squamous cell carcinoma, brain metastases, irradiation, radiation therapy, immunotherapy

## Abstract

The abscopal effect is a phenomenon relating to the treatment of metastatic cancer in which localized irradiation to a tumor concurrently causes shrinkage of tumors distant from the area of treatment. Localized radiotherapy is thought to cause anti-tumor immunologic responses that lead to regression and remission of cancers distant to the initial location of treatment. We present a 47-year-old male with brain metastasis from non-small cell lung cancer (NSCLC) who went into remission following stereotactic radiosurgery treatment to a brain lesion, in the absence of systemic treatment. We discuss the novelty of this case and its importance to future research on the abscopal effect. Though it is difficult to distinguish the abscopal effect from spontaneous remission of non-targeted cancer, this report sheds insight on the potential for improving treatment for the leading cause of cancer death worldwide.

## Introduction

Radiation therapy is a well developed and highly effective form of cancer treatment. It has been primarily used to target localized tumor growth by causing DNA damage within irradiated cells. However, following localized radiation therapy, there have been multiple reports of cancer regression at distant locations. This has been termed the “abscopal” effect [1]. Recent evaluation of the hypothesis suggests the phenomenon is due to systemic immune responses [2-3].

Increased host defenses promoting cell death at distant cancer sites are thought to be a reflection of the immune stimulation provoked by radiotherapy. Triggering of dendritic cell engulfment, augmenting tumor receptors, creating changes in cytokine patterns, and altering expression and functioning of effector T cells are rational mechanistic theories of the immune response from the abscopal effect [2].

Our case demonstrates an atypical response of metastatic lung cancer to radiotherapy, suggesting an abscopal effect. In the absence of systemic treatment, it reinforces the hypothesis that underlying immunomodulation may be an underlying mechanism of the effect.

## Case presentation

Our patient was a 47-year-old male with past medical history including aorto-occlusive disease status post femoral-popliteal bypass, with peripheral artery disease, coronary artery disease, and tobacco dependence. He initially presented with right groin and lower-extremity numbness with an otherwise unremarkable review of systems. The patient was diagnosed with right limb occlusion with critical limb ischemia of the right lower extremity due to an aorto-femoral bypass graft occlusion. Initial workup included a computed tomography (CT) angiogram of the chest, prior to treatment of the occlusion with a femoral-femoral bypass.

Computed tomography angiography (CTA) of the chest revealed a 1.4 cm nodule at the left lung apex, slightly cavitary in nature together with a left paratracheal soft tissue density that was suspected to be adenopathy related to pneumonia that was being treated. The lesion was considered to be incidental with the recommendation of short-term follow-up with another chest CT in three months. There was no prior imaging for comparison.

Two months later, the patient presented to the emergency room with bilateral chest pain and associated shortness of breath and dyspnea. He was admitted to the intensive care unit (ICU) for respiratory instability and treated for multiple bilateral pulmonary embolisms. The diagnosing CTA of the chest showed an increase in the left upper lobe mass density with 2.5 cm x 2.4 cm dimensions including marked interval increase in diffuse mediastinal and bilateral hilar adenopathy involving levels T5, T10, and T11, suggesting a primary neoplasm with metastatic disease. The primary lesion was pleural based and thought to be invading the pleura. Once the patient stabilized, a CT-guided left upper lobe biopsy was obtained.

Biopsy revealed a poorly differentiated non-small cell carcinoma consistent with squamous cell carcinoma. Sections showed nests and individual large cells with brisk mitotic activity with medium to large nuclei. There was considerable tumor necrosis. Immunohistochemical stains showed positive staining for p63 and negative for TTF1. Morphology and stains were consistent with squamous cell carcinoma of the lung. It was suggested that the pulmonary embolisms the patient experienced were attributed to a hypercoagulable state related to malignancy.

**Figure 1:**
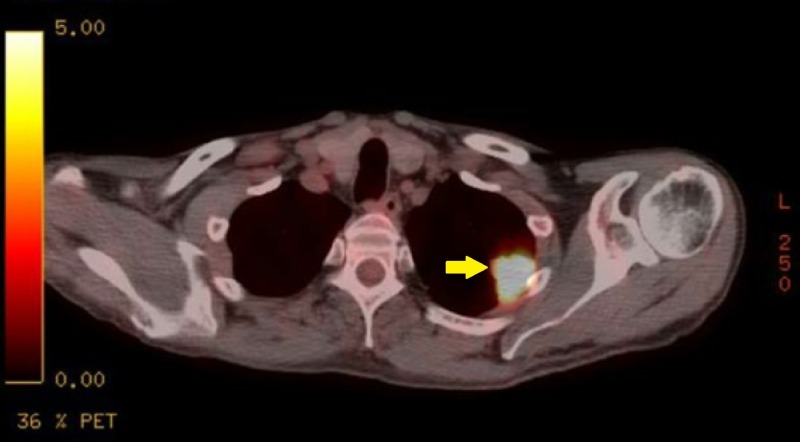
Left Upper Lobe Mass Pre-Treatment Transverse Positron Emission Tomography (PET) Scan. Hypermetabolic left upper lobe lung mass which is pleural-based and potentially invading the pleura.

**Figure 2:**
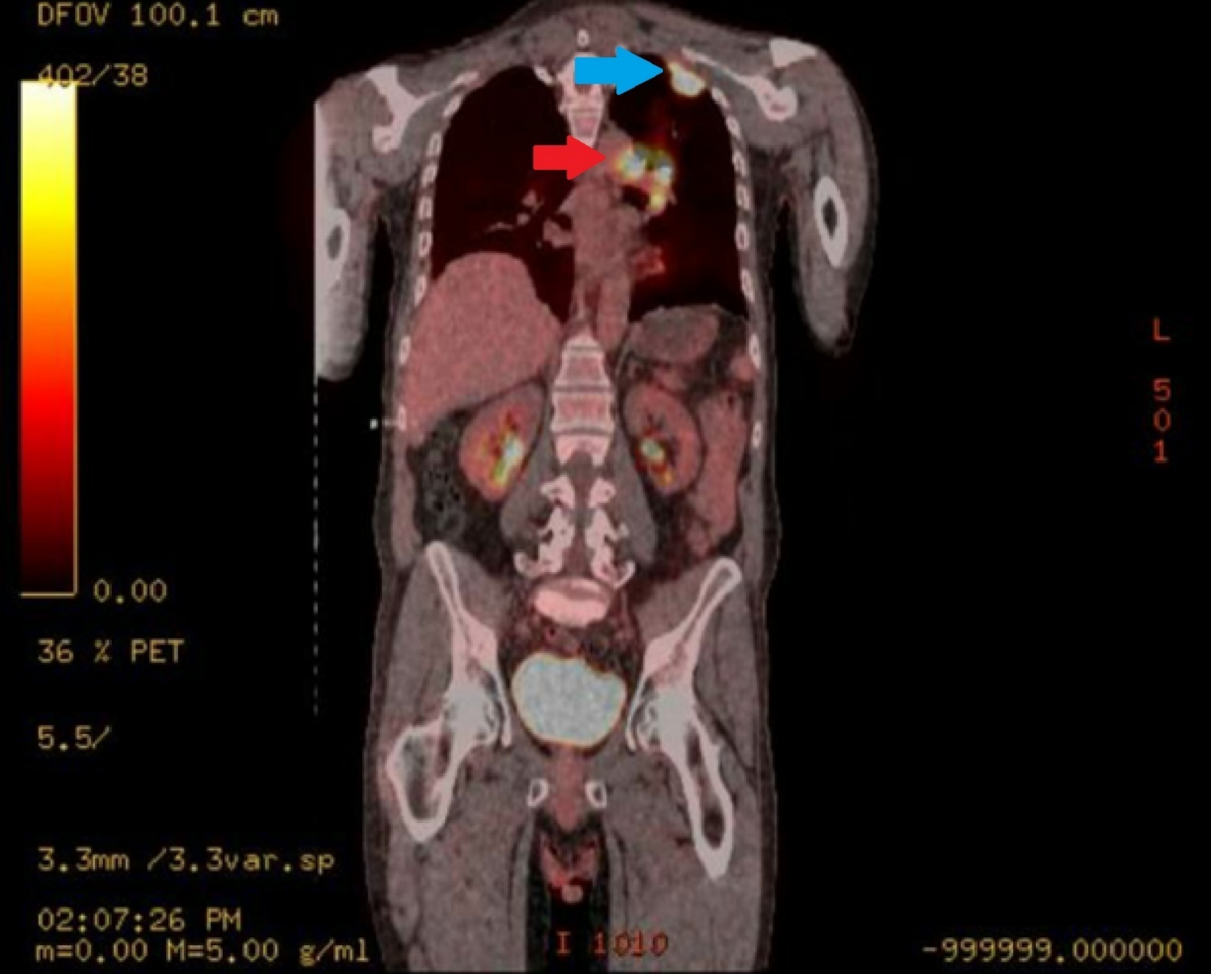
Left Upper Lobe Pre-Treatment Coronal Positron Emission Tomography (PET) Scan. Hypermetabolism appreciated in the left mediastinum (red arrow) and left upper lobe (blue arrow).

**Figure 3:**
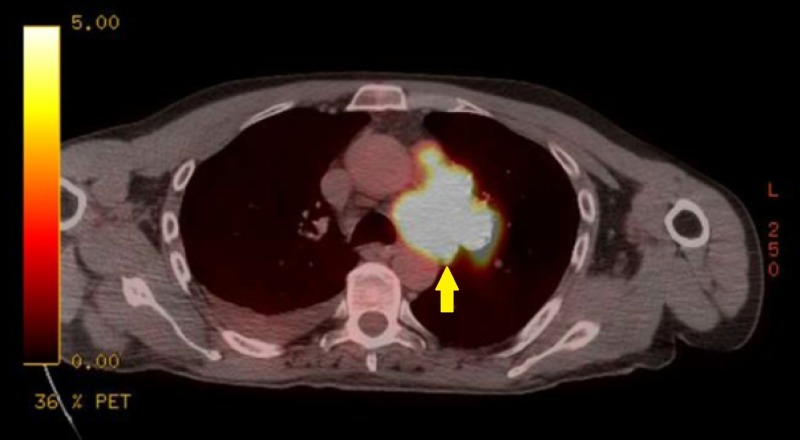
Mediastinal Mass Pre-Treatment Transverse Positron Emission Tomography (PET) Scan. Extensive hypermetabolic left hilar lymphadenopathy, compatible with neoplastic disease and likely metastatic nodal involvement.

**Figure 4:**
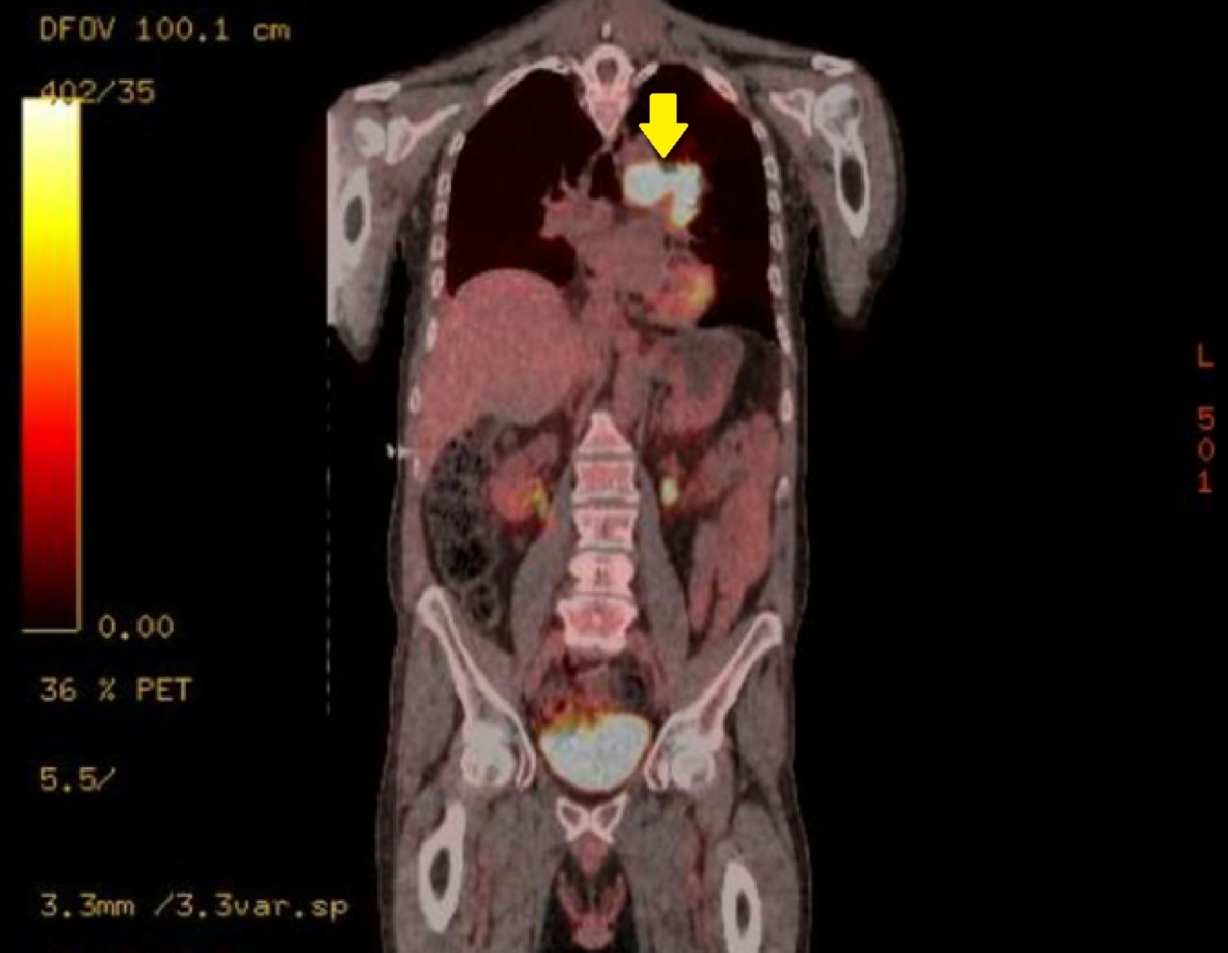
Mediastinal Mass Pre-Treatment Coronal Positron Emission Tomography (PET) Scan.

**Figure 5:**
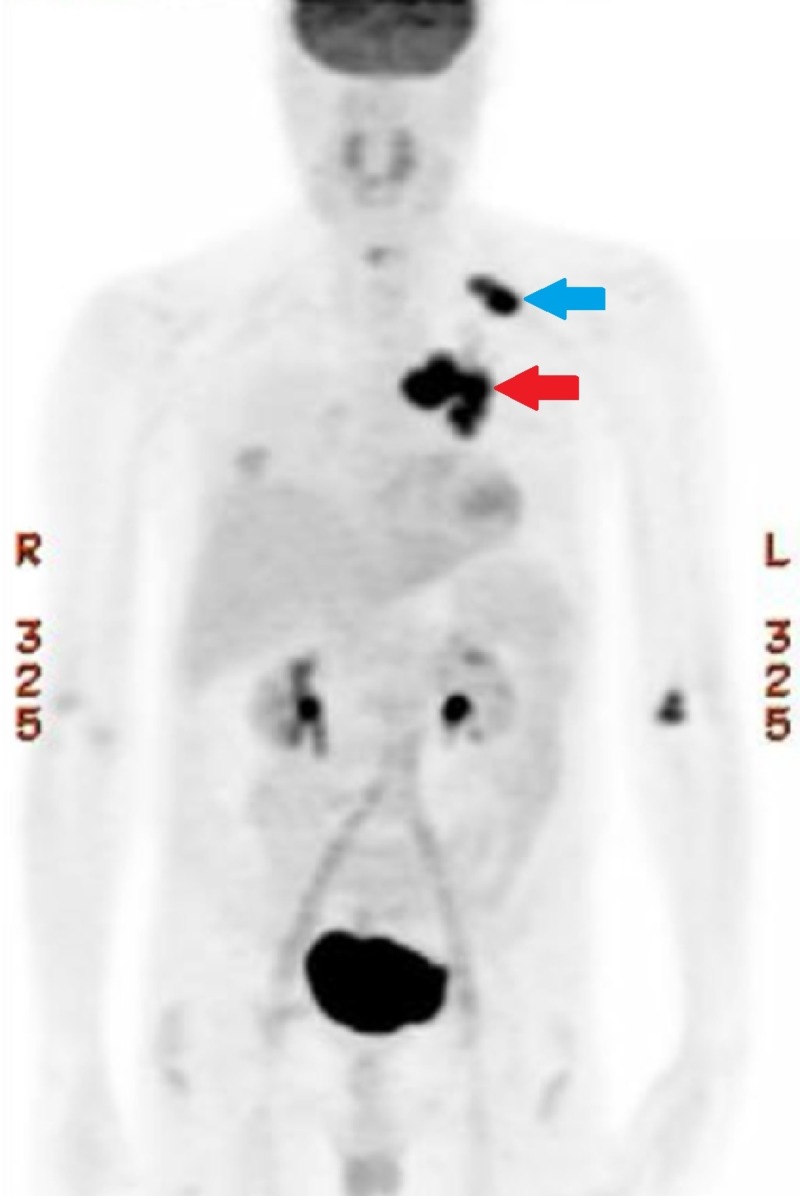
Full Body Pre-Treatment Positron Emission Tomography (PET) Scan. Hypermetabolism appreciated in the left upper lobe (blue arrow) and left mediastinum (red arrow).

**Figure 6:**
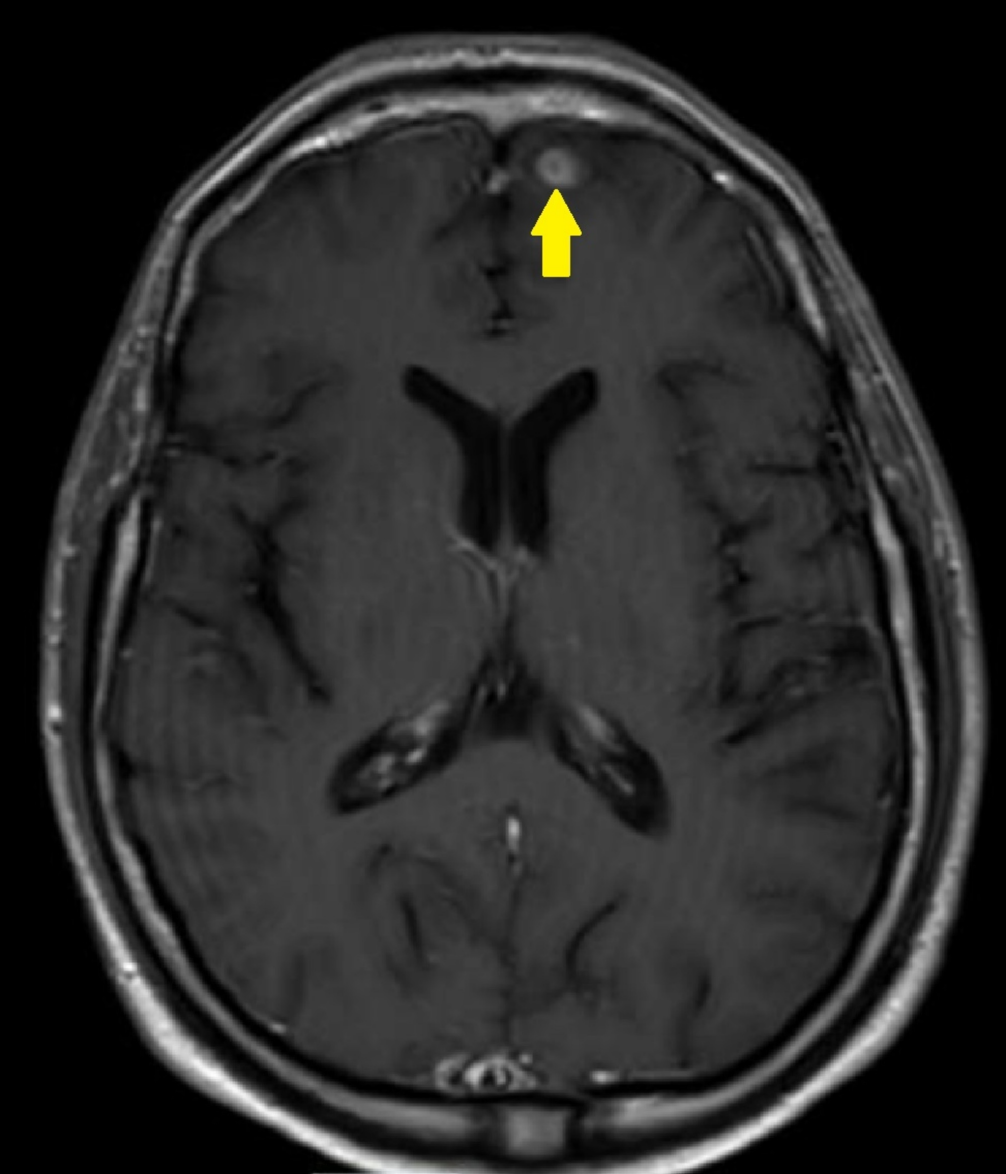
Left Frontal Lobe Metastasis Pre-Treatment Transverse Brain Magnetic Resonance Imaging (MRI). Small 6 mm ring-enhancing metastatic lesion noted in left frontal lobe with some surrounding edematous change.

**Figure 7:**
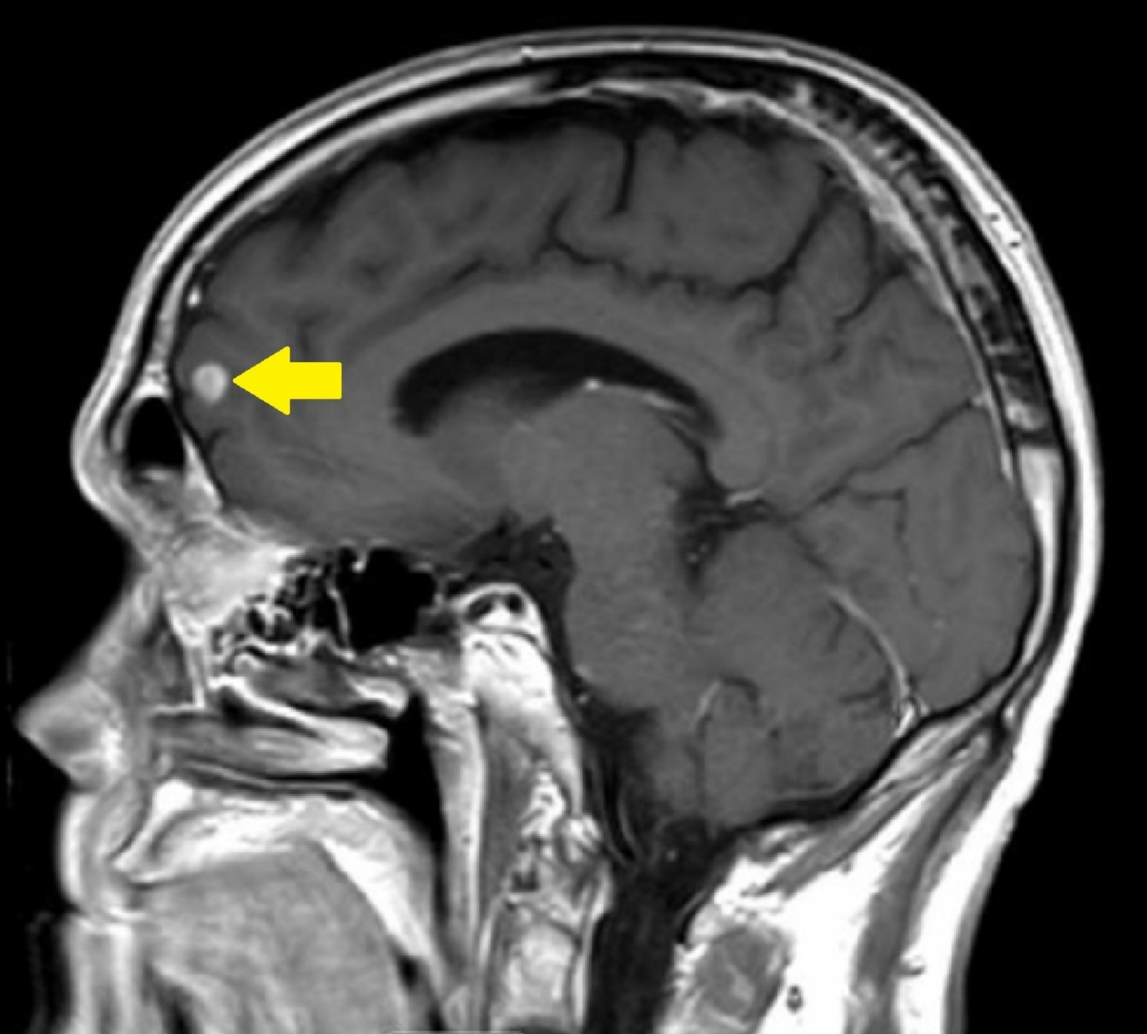
Left Frontal Lobe Metastasis Pre-Treatment Sagittal Brain Magnetic Resonance Imaging (MRI).

Oncologic positron emission tomography (PET)/CT scan suggested invasion of the pleura with perivascular and lymphatic metastatic involvement, confirming a hypermetabolic left upper lobe mass of 2.5 cm x 2.8 cm with SUV of 10.7 (Figures 1-2) and hypermetabolic left hilar adenopathy (Figures 3-5). Subsequently, a magnetic resonance imaging (MRI) of the brain was completed for evaluation of metastasis. A 6 mm ring-enhancing metastatic lesion was noted in the left frontal lobe with surrounding edema (Figures 6-7). The imaging was otherwise unremarkable.

The initial treatment plan included a radiosurgery approach to the solitary brain lesion. The lesion was treated with five volumetric arc therapy (VMAT) beams with the isocenter located in the center of the lesion, which was contoured on the MRI images. A total peripheral dose of 2500 cGy delivered in five fractions was prescribed to the planning target volume (PTV). Dose-volume histogram (DVH) analysis of the target lesions showed dose statistics (minimum, maximum, and mean) of 2396.0 cGy, 2941.2 cGy, and 2759.3 cGy to the PTV, respectively. The total volume of the gross lesion and margins was 0.4 cc. The total volume of tissue receiving 100% of the total prescribed dose (V100%) was 0.5 cc. The patient did not receive any form of systemic therapy, such as chemotherapy or immunotherapy.

**Figure 8:**
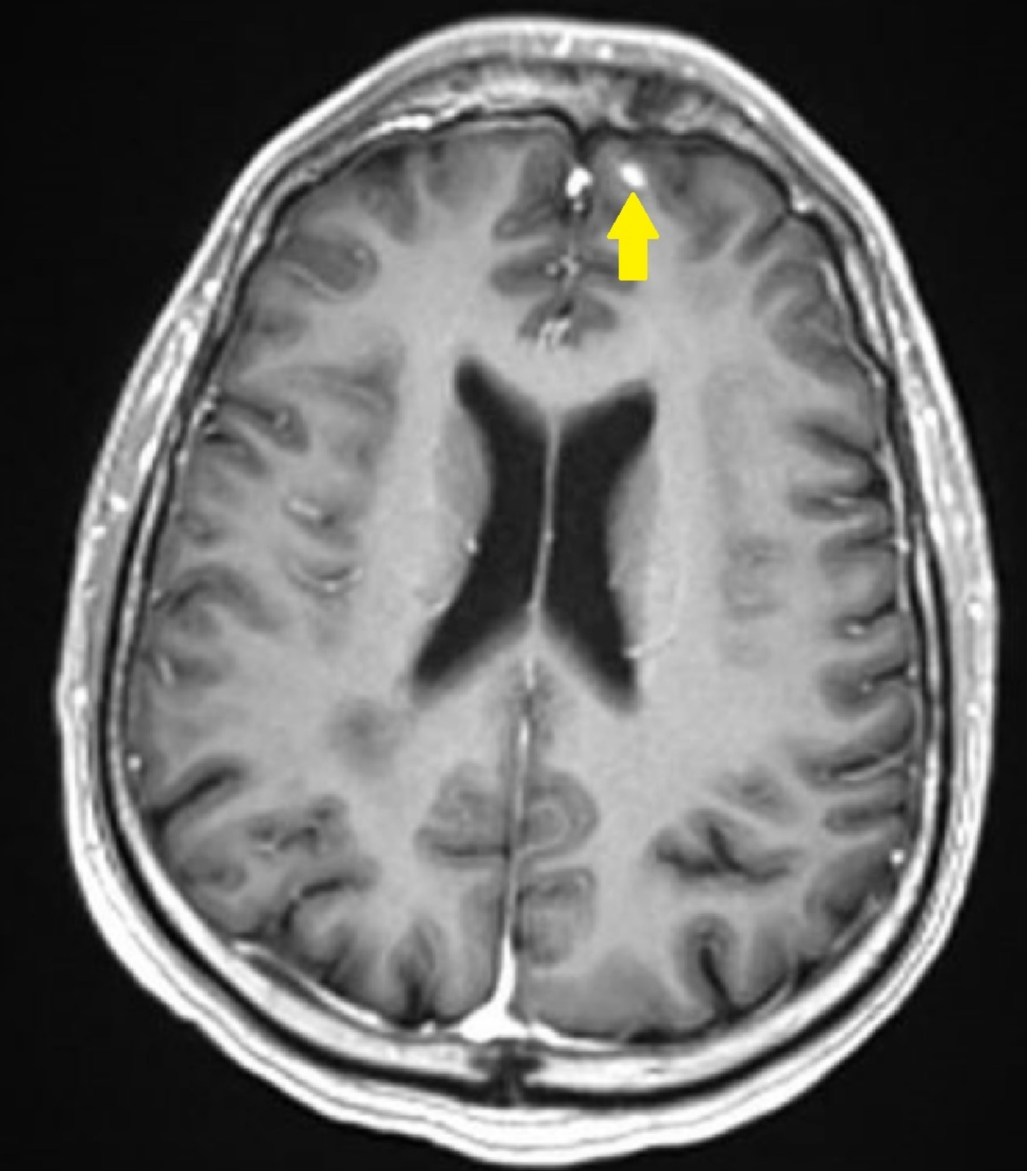
Left Frontal Lobe Metastasis Reduction Post-Treatment Transverse Brain Magnetic Resonance Imaging (MRI). Previously described 6 mm metastatic focus is again seen in the left frontal region appearing smaller.

**Figure 9:**
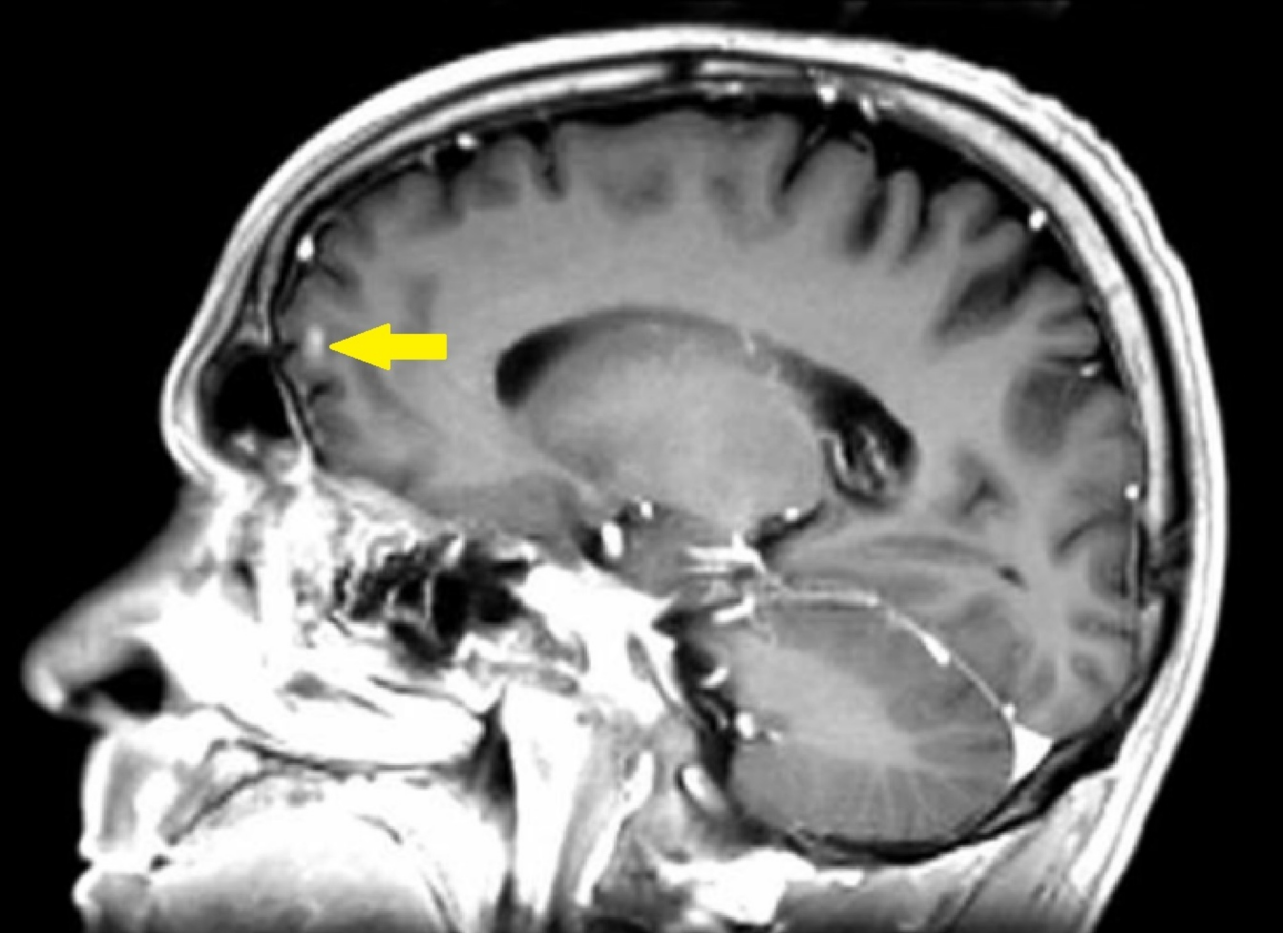
Left Frontal Lobe Metastasis Reduction Post-Treatment Sagittal Brain Magnetic Resonance Imaging (MRI).

**Figure 10:**
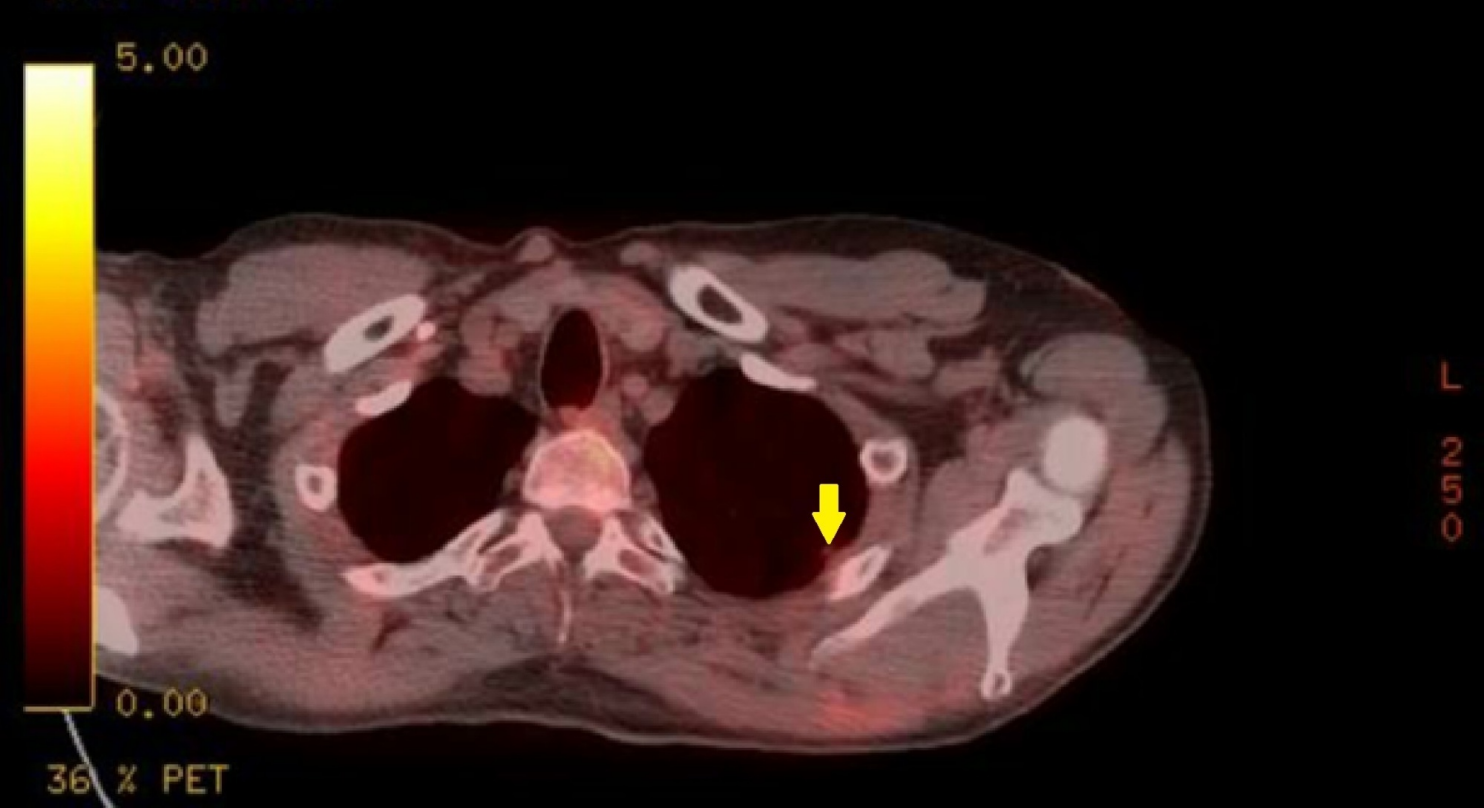
Left Upper Lobe Mass Post-Treatment Transverse Positron Emission Tomography (PET) Scan. Hypermetabolism of left upper lobe mass no longer appreciated.

**Figure 11:**
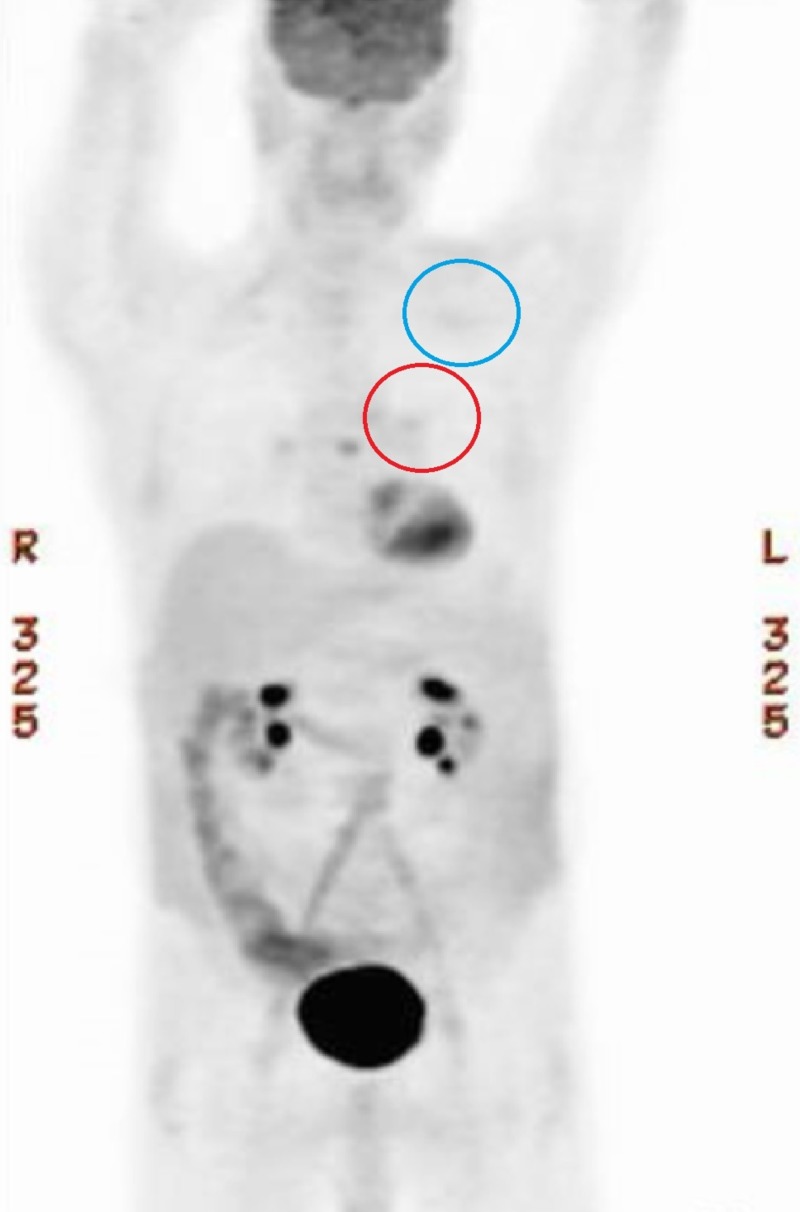
Full Body Post-Treatment Positron Emission Tomography (PET) Scan. Resolution of hypermetabolism in the left mediastinum (red circle) and left upper lobe (blue circle) of the lung.

**Figure 12:**
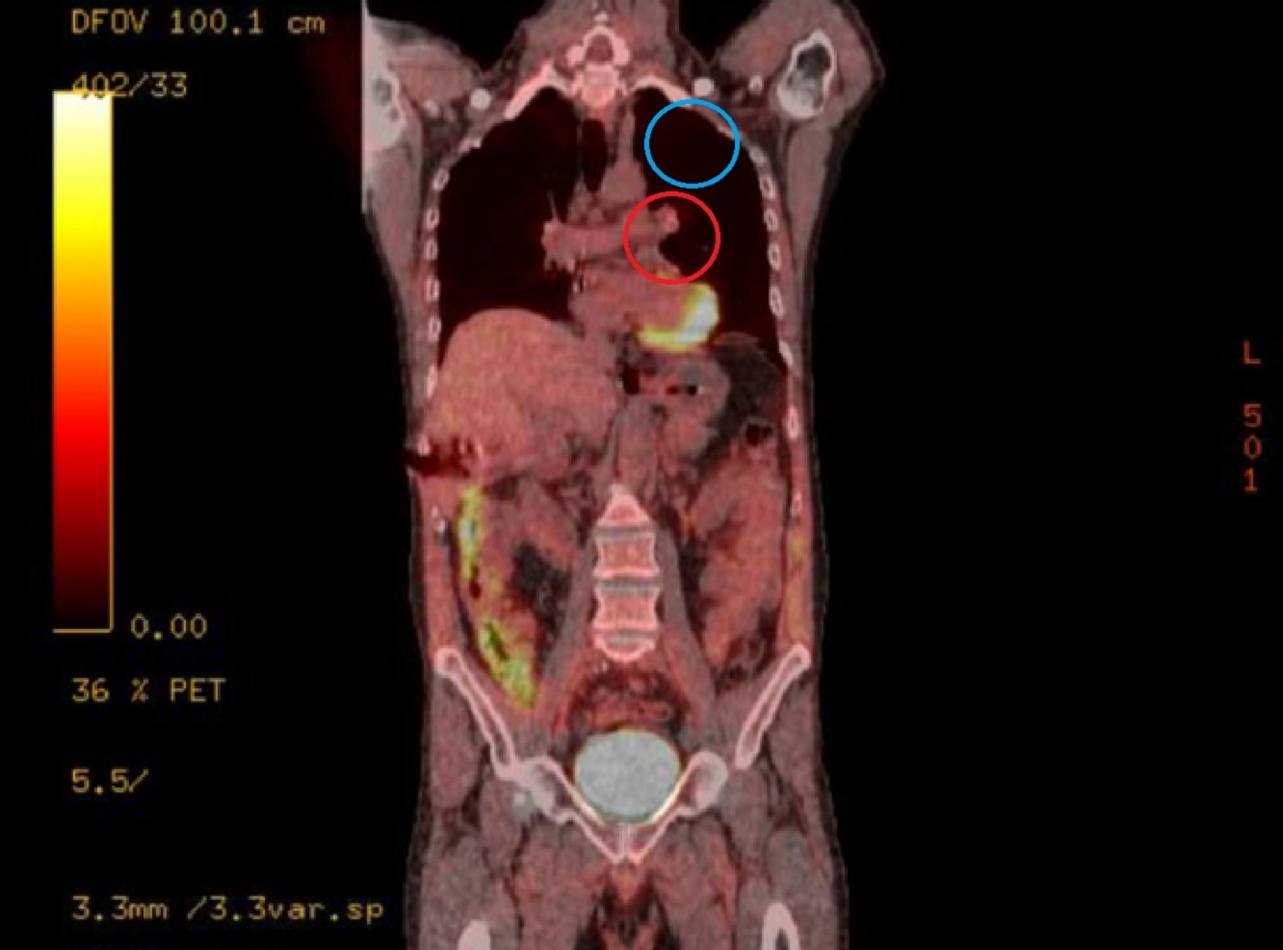
Hypermetabolism Post-Treatment Coronal Positron Emission Tomography (PET) Scan. Resolution of hypermetabolism in the left mediastinum (red circle) and left upper lobe (blue circle) of the lung.

**Figure 13:**
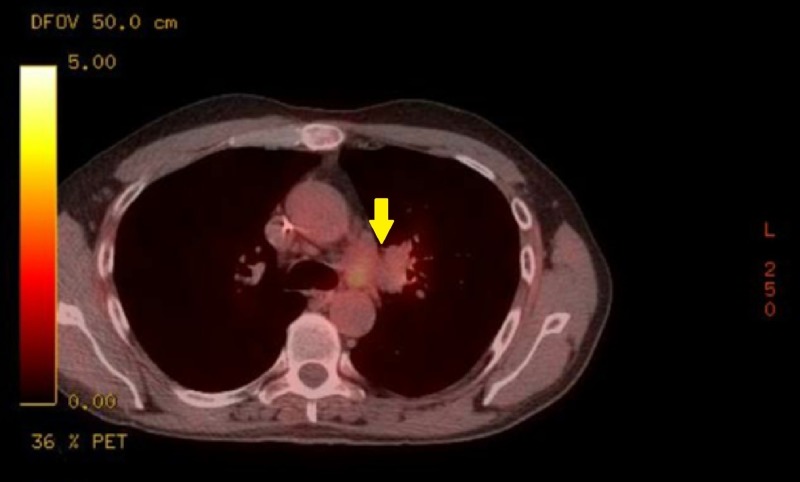
Mediastinal Hypermetabolism Post-Treatment Transverse Positron Emission Tomography (PET) Scan. Fluorodeoxyglucose (FDG) in the left hilum and the left AP window markedly improved with the SUV level 2.2-2.7 compared with the previous 9.3.

Post-treatment MRI of the brain at one-month follow-up after the initial brain lesion diagnosis showed a reduction in size from the original 6 mm nodule (Figures 8-9) on the stealth protocol study. Remarkably, repeat PET/CT four weeks post-treatment stereotactic radiosurgery treatment (SRS) revealed no appreciable mass in the left upper lobe (Figures 10-12) with resolution of hypermetabolism. Fluorodeoxyglucose (FDG) activity in the left hilum gave SUV level 2.2-2.7, an improvement from the previous SUV of 9.3 (Figures 11-13). Chest CT confirmed that there was no longer an appreciable left upper lobe mass. An additional follow-up chest CT was completed two months later (three months post-treatment) confirming complete resolution of the original left upper lobe pleural-based mass.

The most recent imaging at seven months post-treatment continued to remain free of residual left upper lobe and mediastinal masses on chest CT. Brain MRI showed complete resolution of the solitary metastatic focus in the left frontal region. There was no evidence of new metastatic disease.

## Discussion

Though rare, the abscopal effect has become an increasingly documented phenomenon of anti-tumor immune response to radiotherapy. This is a novel case because although the patient’s outcome follows typical abscopal expectations, to our knowledge, this particular combination of cancer and metastasis has yet to be documented and enforces the magnitude that the immune system is able to modulate itself due to the stress of radiotherapy.

This is the first reported abscopal case of a remission of squamous cell carcinoma of the lung following brain metastasis. There was a complete response of the left upper lobe and mediastinal masses following irradiation treatment based on response evaluation criteria of solid tumors (RECIST). No adjuvant systemic treatment was given. It was previously suggested that the abscopal effect would not be appreciated in cases where metastasis crossed the blood-brain barrier [4]. This was based on the observation of the regression of lung and mediastinal disease following radiotherapy, yet with the progression of brain metastasis. The current case, however, contradicts this theory, as the radiosurgery to the brain lesion in this patient appears to have induced remission of the lung cancer. This merely suggests that the inciting mechanisms that elicit the abscopal effect can cross the blood-brain barrier. Multifactorial immune stimulation is likely central to the success of the complete remission of cancer growth.

The majority of reported cases of the abscopal effect are related to traditionally immunogenic diseases: renal cell carcinoma, melanoma, and lymphoma [5]. Despite the majority of these reports, the abscopal effect has been reported across a wide variety of cancers. This suggests that various mechanisms of action may be responsible for creating the distant regression observed, and that radiotherapy has a role in a multitude of disease processes for inducing immune stimulation. It also proposes the thought that immune response leading to cancer remission is more likely to be reported in those diseases that are already known to be immunologic [2]. Due to lack of awareness, or altered perception, reports observing the abscopal effect could be underreported and under-recognized, as differentiating these effects from spontaneous regression is not straightforward.

The abscopal effect is thought to be immune-mediated [3,6]. Although the clinical role of radiation therapy in the treatment of lung cancer combined with traditional chemotherapy is well established, the immune response related to radiation remains relatively poorly understood. If radiation immune mechanisms could be demonstrated to act reliably at a distance, it would represent an important step forward. Although significant incremental improvements in survival have been achieved with chemoradiation protocols, the combination of chemoradiotherapy with immunotherapy is just beginning to be explored. It has now been shown in clinical trials that by using concurrent chemotherapy and sequential treatment with immune modulators, there is greater effectiveness of radiotherapy for lung cancer. The PACIFIC trial in particular demonstrated that adding treatment with the programmed death ligand (PD-L1) inhibitor, Durvalumab, as an adjuvant to chemotherapy and radiation to non-small cell lung cancer (NSCLC) resulted in a longer progression-free survival period and a lower incidence of new brain metastases [7].

There may be theoretical rationale to account for stimulation of the immune system by radiation therapy. Certainly radiation may alter the antigenicity of the tumor cell, altering the tumor microenvironment and potentiating the effect of immune modulation and recruitment of effector T-cells. Radiation cell killing has mainly been ascribed to apoptosis and mitotic catastrophe [8], which could by itself account for changes in antigenicity. The DNA damage response to radiation is likely to induce differing signaling cascades depending on the presence of p53 [9] with possible immunomodulating effects. Other pre-clinical data suggest that radiation may have unrecognized roles in enhancing epithelial-to-mesenchymal cell transition, invasion, migration, angiogenesis, and metastasis [10]. In a mouse model of breast and colon cancer, it was shown that localized primary tumor irradiation together with blockade of CTLA-4 induced an abscopal effect outside the radiation field [6]. The frequency of CD8+ T cells showing tumor-specific interferon (IFN)-gamma production was proportional to the inhibition of the secondary tumor [6].

## Conclusions

The hypothesis that the abscopal effect is due to increased systemic immune modulation from localized irradiation has potential to become clinically relevant and optimize the standard of care for the treatment of NSCLC with metastasis. The current case demonstrates essentially spontaneous regression of significant lung disease after radiosurgery to a small brain metastasis. Ultimately the goal is to improve the future standard of care treatment for patients with NSCLC by reducing both morbidity and mortality.

The clinical course of our patient aligns with previous reported cases recognizing the abscopal effect and further enforces possible immune mechanisms that may aid in the understanding of the abscopal effect, including the ability to cross the blood-brain barrier, the ability for irradiation treatment to overcome potential deleterious effects, and its ability to heal cancers with high morbidity and mortality, including lung cancer with brain metastasis. Because of the uncertainty in the degree of abscopal effect significance, it is important to continue to encourage reporting of post-radiation cancer regressions and remissions as well as continue to study the effects of radiation dosing, timing, and variability in the disease response. As irradiation treatment causes an immune response, it is thought that radiosurgery in conjunction with immunomodulators may potentiate the abscopal effect. Our patient’s case reinforces the potential for making the abscopal effect clinically relevant, and may guide future standard of care for metastasis of the deadliest cancer worldwide. Perhaps in the future, the immune system could reliably be stimulated at a distance from the irradiated target. The abscopal effect seems to be giving us another clue to the puzzle.
